# Graph convolution network based on meta-paths and mutual information for drug-target interaction prediction

**DOI:** 10.1186/s12859-025-06295-x

**Published:** 2025-11-07

**Authors:** Shujuan Cao, Binying Cai, Zhejian Qiu, Tiantian Chang, Qiqige Wuyun, Fang-Xiang Wu

**Affiliations:** 1 School of Mathematical Sciences, Tiangong University, Tianjin, China; 2 School of Science, Xi’an University of Posts and Communications, Xi’an, China; 3 Department of Computer Science and Engineering, Michigan State University, East Lansing, MI USA; 4 Division of Biomedical Engineering, Department of Mechanical Engineering and Department of Computer Science, University of Saskatchewan, Saskatoon, SK Canada

**Keywords:** Graph convolutional networks, Heterogeneous network, Drug-target interaction, Mutual information

## Abstract

**Background:**

Predicting drug-target interactions (DTIs) plays a pivotal role in accelerating drug repositioning by prioritizing candidate drugs and reducing experimental costs. Despite advancements in deep learning, several challenges still require further exploration, including sparsity and inadequate representation of feature relationships.

**Results:**

We propose GCNMM, a novel graph convolutional network based on meta-paths and mutual information, to predict latent DTIs in drug-target heterogeneous networks. Our approach begins by constructing a fused DTI network based on meta-paths and a graph attention network. We compute multiple similarity networks by using Jaccard coefficients and integrate them into the fused drug and target similarity networks through entropy-based fusion. These networks are then jointly processed by graph convolutional auto-encoder to generate low-dimensional feature representations. To preserve the topological structure of the original network in the embedding space and strengthen the relationship between the input and latent representations, we incorporate spatial topological consistency and mutual information maximization as dual optimization objectives.

**Conclusions:**

The experimental results illustrate that GCNMM exhibits superior performance to existing baseline models in DTI prediction. Furthermore, case studies validate the practical effectiveness of GCNMM, highlighting its potential in DTI prediction and drug repositioning.

**Supplementary Information:**

The online version contains supplementary material available at 10.1186/s12859-025-06295-x.

## Introduction

The drug discovery process is time-consuming and expensive, often accompanied by a high failure rate before the market approval. With the growing availability of biological databases and the advances in computational techniques, drug repositioning has emerged as an efficient strategy and a key research focus in pharmaceutical science [1, 2]. By leveraging public databases and bioinformatics methods, researchers can predict high-confidence drug-target interactions (DTIs), thereby narrowing the search space of potential drugs targeting specific diseases or uncovering candidate targets for existing drugs [3, 4].

Existing methods for predicting DTIs mainly include ligand-based methods, structure-based methods, and machine learning methods. However, the first two approaches face significant limitations. Ligand-based methods fail to provide a deep understanding of the mechanisms underlying DTIs [5]. The applicability of structure-based methods is strictly limited to cases where detailed structural information of both drugs and proteins is available [6].

Machine learning approaches have recently gained attention as promising techniques for candidate DTI prediction. These methods are generally partitioned into feature-based methods and network-based methods [7]. For the feature-based methods, Wang et al. integrated pharmacological information, therapeutic effects, molecular structures of drugs, and genomic data of proteins through kernel functions in support vector machine (SVM) to characterize DTIs [8]. Lan et al. developed PUDT that using Random walk with restarts, KNN and heat kernel diffusion to predict DTIs [9]. Yan et al. constructed comprehensive similarity correction matrices for drugs and targets by combining multiple kernel learning with clustering techniques, and subsequently applied a bi-random walk algorithm to infer candidate DTIs [10]. Yu et al. developed FPSC-DTI, a model that combined feature projection fuzzy classification with super cluster classification for the prediction of DTIs [11]. Additionally, a matrix tri-factorization method named NMTF-DTI, employed multiple kernels as similarity metrics and incorporated Laplacian-based regularization to improve DTI prediction accuracy [12]. However, these methods often required substantial prior knowledge and struggled to capture the deeper associations between drugs and targets. By comparison, network-based methods utilized principles of network theory to build heterogeneous networks by incorporating multimodal biological entities. The method NRWRH imported the random walk with restart on a heterogeneous network to predict candidate DTIs [13]. The DTI prediction was framed as a link prediction issue in some studies [14–16]. DTiGEMS + constructed a heterogeneous network by using similarity fusion algorithm, and built a link prediction model of DTIs based on the graph embedded method node2vec and graph mining [14]. Shao et al. introduced DTI-HETA, the model combined Graph Convolutional Neural Network (GCN) with the Graph Attention Mechanism (GAT) to obtain the embedded representations from a heterogeneous network [15]. Fu et al. developed MVGCN, a multi-view link prediction framework that constructed a heterogeneous network and designed an embedding layer with neighborhood information aggregation to enhance the node embedding updates [16]. Wang et al. proposed DTI-BGCGCN which employs a bipartite attribute graph and ClusterGCN to predict DTIs for modern and traditional medicine [17].

Meta-paths are critical constructs in heterogeneous networks, describing composite relationships and capturing underlying semantic information among different nodes. Distinct meta-paths enable the identification of diverse and meaningful relationships among nodes [18]. For instance, Wang et al. [19] applied attention mechanisms at both the node and semantic level to evaluate the influence of nodes and their neighbors defined by meta-paths, as well as the significance of the meta-paths themselves. A new graph neural network framework, MHGNN [20], was developed to capture the complex graph structure and higher-order semantic information by leveraging meta-paths to predict DTIs. Qiao et al. presented DMHGNN [21], which leveraged a graph encoder based on meta-paths combined with a denoising auto-encoder to learn heterogeneous network features, and captured the latent representation of drug-protein pairs (DPPs) via a multi-channel graph convolutional network for predicting DTIs.

Moreover, mutual information (MI) was used to quantify the statistical dependence between two random variables. It has been widely applied in tasks such as feature selection and data fusion. However, traditional techniques for calculating MI encountered substantial difficulties when handling high-dimensional data, including high computational complexity and sensitivity to data distribution [22]. To overcome these limitations, Mutual Information Neural Estimation (MINE) [23] was proposed, offering linear scalability with respect to both dimensionality and sample size. MINE transforms the mutual information estimation problem into an optimization task for neural networks by leveraging gradient descent. Furthermore, Hjelm et al. [24] proposed an unsupervised framework called Deep InfoMax (DIM) that enhanced the representation learning through the joint maximization of mutual information at both the global and local aspects between input data and encoder outputs, while adversarial learning constrains the representations to match a prior distribution.

In this study, a Graph Convolution Network framework based on Meta-paths and Mutual Information, named GCNMM, is proposed to predict potential DTIs by leveraging structural and semantic information in a heterogeneous network. First, indirect DTI networks are constructed through meta-paths, and a fused DTI network is obtained based on meta-path networks and GAT. This process aims to reduce the sparsity of the original DTI network. Similarity networks for drugs and targets are constructed by calculating Jaccard coefficients from multiple aspects, and then fused based on entropy. Second, based on the fused similarity networks and the fused DTI network, the low-dimensional feature vectors are learned with a graph convolutional auto-encoder. During the encoding process, the spatial topological consistency is integrated to ensure the preservation of node topology relationships between the embedding space and the original space as much as possible. Additionally, the MI estimation using global–local-prior discriminators is introduced to further enhance the dependence between the input and outputs of the self-encoder. Finally, an XGBoost classifier is leveraged to predict unobserved DTIs.

The core contributions of GCNMM include the following:

- The fused DTI network based on meta-paths is proposed to reduce the sparsity of the original DTI network and alleviate the imbalance between positive and negative samples.
- Spatial topological consistency is applied to ensure that the nearest-neighbor relationships in the embedded space remain close to the original space as much as possible.
- Estimations of global, local, and prior mutual information are incorporated to strengthen the correlations between the fused DTI network and the feature vectors.
- Experimental results show that GCNMM consistently superior to the baseline methods for DTI prediction. Furthermore, the effectiveness and robustness of the model are thoroughly validated through ablation experiments and case studies.

## Preliminaries

This part provides formal definitions relevant to heterogeneous networks.

Let $G=\left(V,E\right)$ denote a graph, where $V$ represents the node set, $E$ denotes the edge set. A heterogeneous graph [18] is also linked to a node category mapping function $A' \to A$, and an edge category mapping function $B' \to B$, $A$ and $B$ represent the quantities of node and edge categories, where $\left|A\right|+\left|B\right|>2$.

**Fig. 1:**
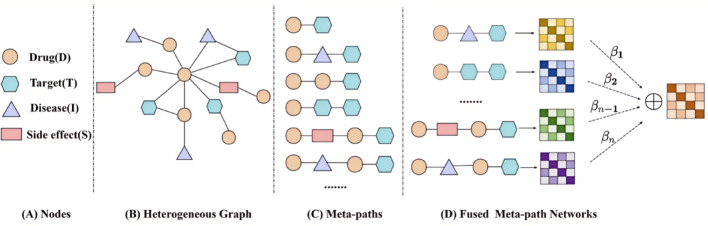
An illustration of a fused DTI network. (**A**) Node types (i.e., drug, target, disease, side effect). (**B**) A heterogeneous graph. (**C**) Meta-paths extracted from a heterogeneous graph. (**D**) A fused meta-path network based on GAT

In our model, a heterogeneous graph is built to depict correlations among bio-entities, including Target (T), Drug (D), Disease (I), and Side effect (S). Edges represent the interactions among bio-entities, such as D-T, D-D, T-I, T-T, shows in Fig. 1(A)-(B).

The meta-path [18] $M$ can be described as ${A}_{1}\stackrel{{B}_{1}}{\to }{A}_{2}\stackrel{{B}_{2}}{\to }\cdots \stackrel{{B}_{l}}{\to }{A}_{l+1}$, where node categories ${A}_{1},{A}_{2},\cdots ,{A}_{l+1}\in A$, edge categories ${B}_{1},{B}_{2},\cdots ,{B}_{l+1}\in B$. Nodes ${A}_{1}$ and ${A}_{l+1}$ are connected by a composite relation ${B}_{1}\circ {B}_{2}\circ \cdots \circ {B}_{l}$, where $\circ$ denotes a composite operator on the connection relation.

Meta-paths are capable of capturing specific semantic information within a heterogeneous graph, as distinct meta-paths convey different semantics. Particularly, as shown in Fig. 1(C), there are multiple meta-paths, D-T shows the known drug-target interactions, D-I-T indicates a drug-target pair interconnected through the common disease, D-D-T represents a drug-target pair connected with the same drug.

A given meta-path instance ${M}_{i,j}$ is a chain of nodes in the heterogeneous graph that follows the definition of the meta-path, $i$ and $j$ correspond to the initial and terminal nodes of the meta-path, respectively. For example, D_1_-I_1_-T_1_, D_1_-I_2_-T_1_, D_2_-I_2_-T_1_ and D_2_-I_2_-T_2_ are four distinct instances that satisfy the meta-path D-I-T.

## Method

**Fig. 2:**
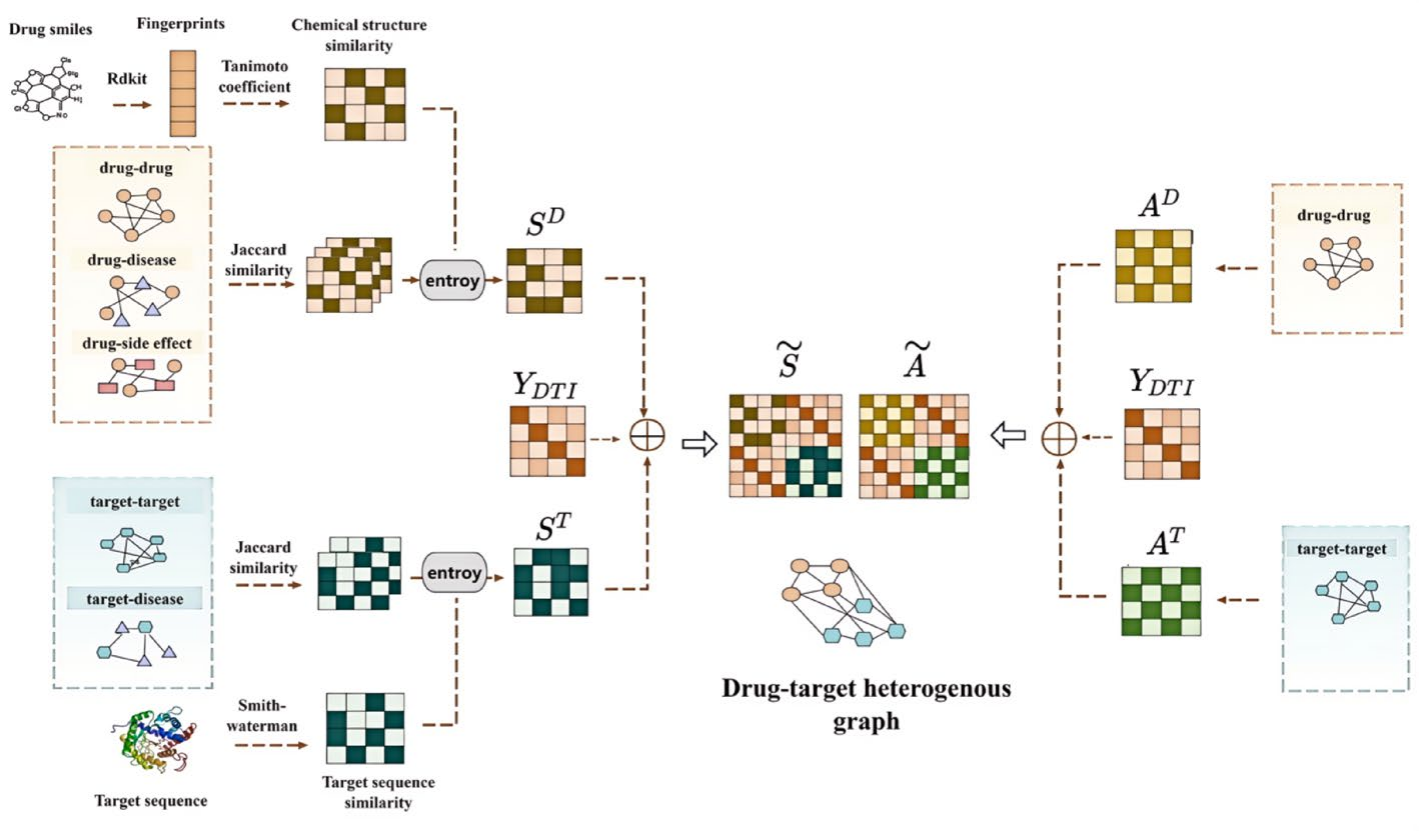
The scheme of Network Fusion. The fused similarity network $\widetilde{S}$ are obtained by concatenating the drug fused similarity network ${S}^{D}$, the target fused similarity network ${S}^{T}$ and the fused meta-path network ${Y}_{DTI}$. The fused interaction heterogeneous network $\widetilde{A}$ are obtained by concatenating the drug-drug interaction network ${A}^{D}$, the target-target interaction network ${A}^{T}$ and the fused meta-path network ${Y}_{DTI}$

**Fig. 3:**
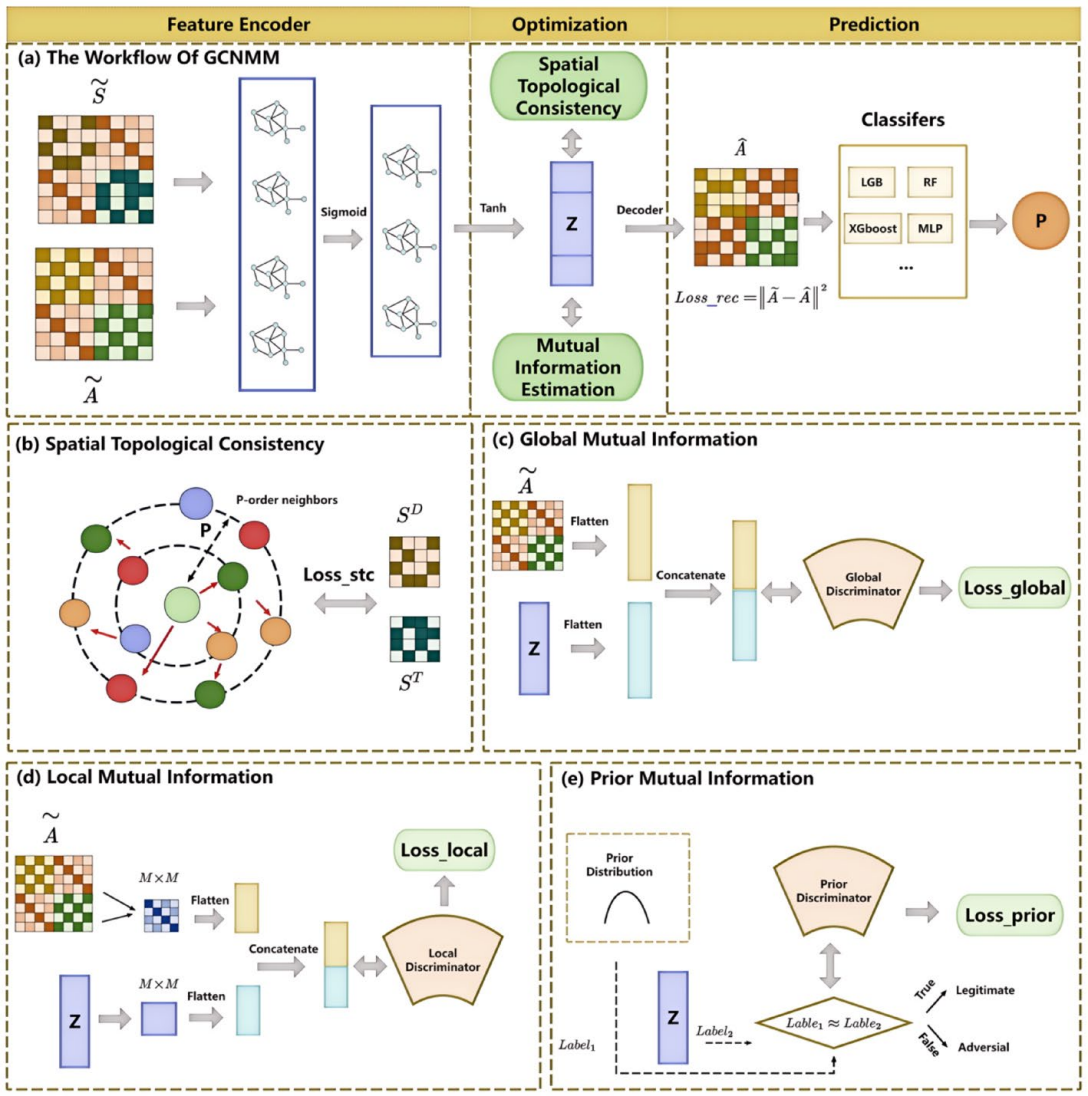
Illustration of GCNMM. (**a**) The workflow of GCNMM includes feature encoder, optimization and prediction module. (**b**) Architecture of spatial topological consistency. (**c**)-(**d**) Architecture of global, local and prior mutual information

In this section, we present an innovative GCN-based approach (GCNMM) to predict candidate DTIs. As depicted in Figs. 2 and 3, the overall workflow of GCNMM consists of feature encoder module, optimization module, and prediction module.

### Meta-path networks

Based on previous studies [25–27], we obtain six networks: Drug-drug interaction network ${A}_{drug-drug}$, drug-target interaction network ${A}_{drug-target}$, drug-disease interaction network ${A}_{drug-disease}$, drug-side effect interaction network ${A}_{drug-side effect}$,

,target-target interaction network ${A}_{target-target}$, and target-disease interaction network ${A}_{target-disease}$, if a node $i$ and a node $j$ have a known association, the item ${A}_{i,j}=1$, otherwise, ${A}_{i,j}=0$.

In the original dataset, just a minor proportion of drug-target pairs exhibit known interactions, while the majority remain unexplored. As a result, many isolated nodes appear in the networks, making it difficult for GCN-based models to effectively capture their characteristics [28]. To address this limitation, we introduce meta-paths to uncover latent interactions between disconnected nodes. Each meta-path is constrained to start from a drug and end at a target, and its length is limited to no more than 5 [29], which is sufficient to retain meaningful semantic and structural information.

We obtain nine distinct meta-paths {DT, DDT, DIT, DTT, DIDT, DITT, DSDT, DDIT, DTDT}, the corresponding networks are denoted as $\left\{{A}_{1},{A}_{2},\cdots ,{A}_{9}\right\}$. For example, the indirect DT association matrix ${A}_{DIDT}$ can be obtained by integrating drug-disease association information and direct drug-target association information, the formula is below: ${A}_{DIDT}={A}_{DI}*{A}_{DI}^{T}*{A}_{DT}$.

#### Fused meta-path network based on GAT

The meta-path D-T denotes the direct drug-target interaction, how to effectively fuse different indirect drug-target interactions is a core issue to be addressed. The GAT [30] is introduced to capture latent semantic significance and efficiently aggregate the information encapsulated within the meta-paths of indirect drug-target interactions, as shown in Fig. 1(D).

To effectively learn the features, a linear transformation is applied to project the network into a uniform embedding space, then we employ GAT to compute attention weights ${w}_{i}$ and obtain fused meta-path network ${A}_{pat{h}_{-}fusion}$:

$${z}_{i}={f}_{\theta }\left({A}_{i}\right)={W}_{1}\cdot {A}_{i}+{b}_{1}, i=2,3,\cdots ,9.$$

$${w}_{i}=q\cdot \text{tanh}\left({W}_{2}\cdot {z}_{i}+{b}_{2}\right),$$

1 $${A}_{pat{h}_{-}fusion}=\sum_{i=2}^{9}{A}_{i}\cdot soft\text{max}\left({w}_{i}\right).$$

where ${f}_{\theta }\left(\cdot \right)$ denotes the linear projection function, ${z}_{i}$ denotes the projected feature, $q$ refers to the learnable attention vector, ${W}_{j}$ refer to the learnable weight matrices, ${b}_{j}$ refer to the learnable bias vectors, respectively. $soft\text{max}\left(\cdot \right)$ is a softmax activation function for normalizing attention weights.

The elements in the ${A}_{pat{h}_{-}fusion}$ represent the magnitude of interactions, the higher values indicate stronger indirect DTIs. A threshold value is applied to discretize the network:

2 $$ Y_{{DTI}}^{{meta}} = ~\left\{ {\begin{array}{*{20}c} {0, A_{{ij}} < \tau ,} \\ {1, A_{{ij}} \tau .} \\ \end{array} } \right. $$

where $\tau$ is a predefined threshold value.

The final fused meta-path network ${Y}_{DTI}$ is obtained as follows:

3 $${Y}_{DTI}=\text{max}\left({A}_{1},{Y}_{DTI}^{meta}\right).$$

### Multiple similarities fusion

#### Drug chemical similarity

RDKit is used to convert Simplified Molecular Input Line Entry Specification (SMILES) [31] sequences of drugs into corresponding Morgan fingerprints [32], subsequently the chemical similarity between drug molecules is quantified by means of the Tanimoto coefficient:

4 $$T\left({r}_{i},{r}_{j}\right)=\frac{{f}_{{r}_{i}}{f}_{{r}_{j}}}{{\Vert {f}_{{r}_{i}}\Vert }^{2}+{\Vert {f}_{{r}_{j}}\Vert }^{2}-{f}_{{r}_{i}}{f}_{{r}_{j}}}.$$

where ${f}_{{r}_{i}}$ refers to the Morgan fingerprints of drug ${r}_{i}$. The drug chemical similarity network is constructed, denoted as ${S}_{chemical}^{D}$.

#### Target sequence similarity

For target sequence, the Smith-Waterman algorithm [33] is applied to compute pairwise similarity, followed by a normalization process:

5 $${S}_{sequence}^{T}\left(i,j\right)=\frac{sw\left(i,j\right)-\text{min}\left(s{w}_{i}\right)}{\text{max}\left(s{w}_{i}\right)-\text{min}\left(s{w}_{i}\right)}.$$

where $sw\left(i,j\right)$ is the sequence similarity score between targets $i$ and $j$, $s{w}_{i}$ indicates the sequence similarity score between target $i$ and other targets, $\text{max}\left(s{w}_{i}\right)$ and $\text{min}\left(s{w}_{i}\right)$ indicate the highest and lowest sequence similarity values between the target $i$ and other targets, respectively. The target sequence similarity network is designated as ${S}_{sequence}^{T}$.

#### Interaction similarity

To accurately capture the structural attributes of the network and thoroughly investigate the latent correlations among nodes, the similarity of interaction network is assessed by using Jaccard similarity.

Jaccard Similarity is an index employed to quantify the degree of similarity between two sets. Given two elements ${x}_{i}$ and ${x}_{j}$ in an interaction network, their Jaccard similarity coefficients are calculated in the following:

6 $$Sim\left(i,j\right)=\frac{\left|N\left({x}_{i}\right)\cap N\left({x}_{j}\right)\right|}{\left|N\left({x}_{i}\right)\cup N\left({x}_{j}\right)\right|},$$

where $\left|N\left({x}_{i}\right)\cap N\left({x}_{j}\right)\right|$ refers to the count of shared neighbors of the two elements, $\left|N\left({x}_{i}\right)\cup N\left({x}_{j}\right)\right|$ represents the total count of neighbors connected to either element ${x}_{i}$ or ${x}_{j}$. The Jaccard similarity coefficient ranges within the interval [0,1], with values proximal to 1 indicating a strong similarity between the elements, and values proximal to 0 indicating a weak similarity.

For D-D network, D-I network, and D-S network, the corresponding three drug-drug similarity networks is obtained: ${S}_{interaction}^{D}$, ${S}_{disease}^{D}$, ${S}_{sider}^{D}$. Similarly, for T-T network and T-I network, their corresponding target-target similarity networks are ${S}_{interaction}^{T}$ and ${S}_{disease}^{T}$.

#### Similarity network fusion

Subsequently, we integrate above similarity networks by leveraging information entropy [34]. Taking drug similarity network ${S}_{sider}^{D}$ as an example, we calculate the entropy of each row in the ${S}_{sider}^{D}$ and calculate their average value:

$${E}_{sider}^{i}=-\sum_{j=1}^{k}\frac{{S}_{sider}^{D}\left(i,j\right)}{\sum_{j=1}^{k}{S}_{sider}^{D}\left(i,j\right)}\text{log}\left(\frac{{S}_{sider}^{D}\left(i,j\right)}{\sum_{j=1}^{k}{S}_{sider}^{D}\left(i,j\right)}\right),$$

7 $${E}_{sider}^{mean}=\sum_{i=1}^{k}\frac{{E}_{sider}^{i}}{k}.$$

where ${S}_{sider}^{D}\left(i,j\right)$ denotes the drug-side effect similarity coefficient, and $k$ denotes the count of rows in a similarity network.

The average entropy of the similarity network is a crucial factor in similarity measures. A similarity network with lower average entropy means the less random information and has a greater weight in a comprehensive similarity network. The weight ${\omega }_{m}$ of drug similarity network $m$ is specified as follows:

8 $${\omega }_{m}=\frac{\raisebox{1ex}{$1$}\!\left/ \!\raisebox{-1ex}{${E}_{m}^{\text{mean}}$}\right.}{\sum_{m=1}^{n}\raisebox{1ex}{$1$}\!\left/ \!\raisebox{-1ex}{${E}_{m}^{mean}$}\right.},$$

where $n$ denotes the number of drug similarity networks.

Finally, the drug fused similarity network is constructed:

9 $${S}^{D}={\omega }_{1}{S}_{\text{interaction}}^{D}+{\omega }_{2}{S}_{\text{disease}}^{D}+{\omega }_{3}{S}_{\text{s}ider}^{D}+{\omega }_{4}{S}_{chemical}^{D}.$$

Similarly, the target fused similarity network is obtained:

10 $${S}^{T}={\eta }_{1}{S}_{\text{interaction}}^{T}+{\eta }_{2}{S}_{\text{disease}}^{T}+{\eta }_{3}{S}_{sequence}^{T},$$

where, ${\eta }_{i}$ is the weight of target similarity network $i$ ($i$=1,2,3).

### Heterogeneous network construction

The set $D=\left\{{d}_{i}\left|i=1,...{N}_{d}\right.\right\}$, ${N}_{d}$ denotes the count of drugs, while another set $T=\left\{{t}_{j}\left|j=1,...{N}_{t}\right.\right\}$, ${N}_{t}$ denotes the count of targets. The fused meta-path network denotes as ${Y}_{DTI}\in {R}^{{N}_{d}\times {N}_{t}}$, if drugs and targets have interactions ${Y}_{DTI}\left(i,j\right)=1$, and otherwise ${Y}_{DTI}\left(i,j\right)=0$. To deeper capture the intrinsic associations and attributes of drugs and targets, two heterogeneous networks are constructed. One is the fused interaction heterogeneous network $\widetilde{A}$, which comprises the drug-drug interaction network ${A}^{D}$, the target-target interaction network ${A}^{T}$ and the ${Y}_{DTI}$, the other is fused similarity network $\widetilde{S}$, which is composed of drug fused similarity network ${S}^{D}$, target fused similarity network ${S}^{T}$ and the ${Y}_{DTI}$. The specific workflow is shown in Fig. 2. The two heterogeneous networks are as follows:

$$\widetilde{A}=\left(\begin{array}{cc}{A}^{D}& {Y}_{DTI}\\ {Y}_{DTI}^{T}& {A}^{T}\end{array}\right) \widetilde{S}=\left(\begin{array}{cc}{S}^{D}& {Y}_{DTI}\\ {Y}_{DTI}^{T}& {S}^{T}\end{array}\right)$$

### Network representation learning

#### GCN encoder

The feature representations of $\widetilde{A}$ and $\widetilde{S}$ are obtained through GCN, as shown in Fig. 3. Specifically, to incorporate the self-information of each node within the network, we set ${A}^{{^{\prime}}}=\widetilde{A}+I$, then ${A}^{{^{\prime}}}$ is normalized to obtain the Laplace matrix. The GCN encoding process for heterogeneous networks is specified as follow:

$${H}^{\left(1\right)}=f\left(\widetilde{S},\widetilde{A}\right)={\sigma }_{1}\left({D}^{-\frac{1}{2}}{A}^{{^{\prime}}}{D}^{-\frac{1}{2}}\widetilde{S}{W}_{\left(1\right)}\right),$$

$${H}^{\left(2\right)}=f\left({H}^{\left(1\right)},\widetilde{A}\right)={\sigma }_{2}\left({D}^{-\frac{1}{2}}{A}^{{^{\prime}}}{D}^{-\frac{1}{2}}{H}^{\left(1\right)}{W}_{\left(2\right)}\right),$$

11 $$D=\text{diag}\left\{{D}_{1},\cdots ,{D}_{\left({N}_{d}+{N}_{t}\right)}\right\},{ D}_{i}=\sum_{j}{A}^{{^{\prime}}}\left(i,j\right).$$

where $D$ is a diagonal matrix, ${H}^{\left(l\right)}$ and ${\sigma }_{l}$ are the $l$-layer embedding of nodes and nonlinear activation function, respectively, where $l=1,2$. Additionally, we set ${\sigma }_{1}=sigmoid\left(t\right)$ and ${\sigma }_{2}=\text{tanh}\left(t\right)$. ${W}_{\left(l\right)}$ represents the weight matrix of the $l$-layer embedding, ${W}_{\left(1\right)}\in {R}^{\left({N}_{d}+{N}_{t}\right)\times m}$ and ${W}_{\left(2\right)}\in {R}^{m\times k}$. After two layers of encoder training, we derive a low-dimensional feature vector $Z\in {R}^{\left({N}_{d}+{N}_{t}\right)\times k}$.

#### Decoder

The vector $Z$ is obtained by performing the encoding process, the reconstructed network $\widehat{A}$ is calculated below:

12 $$\widehat{A}=sigmoid\left(Z\cdot {Z}^{T}\right).$$

The elements of $\widehat{A}$ are the predicted scores of the DTIs. The higher scores correspond to increased association probability.

Next, the Mean Squared Error (MSE) is utilized to measure the deviation between the reconstructed network $\widehat{A}$ and the original heterogeneous network $\widetilde{A}$. The reconstitution loss function takes the following form:

13 $$Los{s}_{reconstitution}={\Vert \widetilde{A}-\widehat{A}\Vert }^{2}={\sum_{i}\sum_{j}\left(\widetilde{A}\left(i,j\right)-\widehat{A}\left(i,j\right)\right)}^{2}.$$

### Optimization

#### Spatial topological consistency

Many potential drug-target interactions remain undiscovered within the network. Relying solely on low-dimensional feature vectors may disrupt their nearest-neighbor relationships in the embedding space, thereby negatively impact on the accuracy of the DTI prediction.

Spatial topological consistency (STC) [35] is mainly to constrain the p-order neighbors of nodes, that is, for any nodes in the original domain, its p neighbors should maintain a close distance in the embedded domain. Therefore, we construct p-nearest neighbor networks for drugs and targets separately. Considering the drug as a case, its p-nearest neighbor network can be obtained by the following formula:

14 $$N\left(i,j\right)=\left\{\begin{array}{c}1,j\in {\mathcal{N}}_{p}\left(i\right),i\in {\mathcal{N}}_{p}\left(j\right),\\ 0,j\notin {\mathcal{N}}_{p}\left(i\right),i\notin {\mathcal{N}}_{p}\left(j\right),\\ 0.5,otherwise.\end{array}\right.$$

We get the drug sparse similarity network ${\widehat{S}}^{D}\left(i,j\right)\in {R}^{{N}_{d}\times {N}_{d}}$ containing the neighbor information as follows:

15 $${\widehat{S}}^{D}\left(i,j\right)=N\left(i,j\right)\cdot {S}^{D}\left(i,j\right).$$

Similarly, after the same steps we get the target sparse similarity network ${\widehat{S}}^{T}\left(i,j\right)\in {R}^{{N}_{t}\times {N}_{t}}$.

Then, the spatial topological consistency loss is delineated by the following expression:

$$Los{s}_{STC}={\lambda }_{1}\left({\Vert {Z}^{D}\Vert }_{F}^{2}+{\Vert {Z}^{T}\Vert }_{F}^{2}\right)$$

$${+\lambda }_{2}\sum_{i,r=1}^{{N}_{d}}{\widehat{S}}^{D}\left(i,r\right){\Vert {Z}_{i}^{D}-{Z}_{r}^{D}\Vert }^{2}$$

16 $$+{\lambda }_{3}\sum_{i,r=1}^{{N}_{t}}{\widehat{S}}^{T}\left(j,q\right){\Vert {Z}_{j}^{T}-{Z}_{q}^{T}\Vert }^{2}.$$

where ${\Vert \cdot \Vert }_{F}$ is Frobenius norms, ${\lambda }_{1}$,${\lambda }_{2}$ and ${\lambda }_{3}$ are nonnegative hyperparameters that determine the relative importance of the three terms. Specifically, the first term serves as a regularization to prevent overfitting. The second term measures the distances among drugs in the embedding space, ensuring that drugs which are close to each other in the original network maintain a similar proximity in the embedding space. Similarly, the third item guarantees that the neighbor information among targets is preserved.

#### Mutual information estimation

MI [22] enables to precisely quantify the degree of associations between variables grounded in Shannon entropy. For example, the MI between variables $X$ and $Z$ can be regarded as the reduction in the uncertainty of $X$ given $Z$:

17 $$I\left(X,Z\right)=H\left(X\right)-H\left(X\left|Z\right.\right),$$

where $H$ denotes the Shannon entropy, $H\left(X\left|Z\right.\right)$ denotes the conditional entropy of $X$ given $Z$.

Similarly, MI can be articulated in light of the Kullback–Leibler (KL) divergence. It is calculated by the KL divergence of the joint probability distributions of $p\left(x\left|z\right.\right)p\left(z\right)$ and $p\left(z\right)p\left(x\right)$:

$${D}_{KL}\left(p\left(x,z\right)\Vert p\left(x\right)p\left(z\right)\right)=\iint p\left(x,z\right)\text{log}\frac{p\left(x,z\right)}{p\left(x\right)p\left(z\right)}dxdz$$

$$=\iint p\left(x\left|z\right.\right)p\left(z\right)\text{log}\frac{p\left(x\left|z\right.\right)}{p\left(x\right)}dzdx$$

18 $$=I\left(X,Z\right),$$

where $p\left(x\right)$ denotes the input distribution, $p\left(z\right)$ denotes the distribution of the output features, $p\left(x,z\right)$ refers to the joint distribution of the input and the output features. Obviously, the greater the discrepancy between the joint probability distribution $p\left(x,z\right)$ and marginal probability distributions' product $p\left(z\right)p\left(x\right)$, the stronger the dependence between $X$ and $Z$. When the divergence is 0, $X$ and $Z$ are independent of each other.

Furthermore, we conduct mutual information estimation through global, local and prior mutual information estimation to optimize the encoder's performance by leveraging diverse objectives and features.

The objective of global mutual information estimation is concentrating on the entire input and output features. At a macroscopic level, it assesses the model's retention of the input network structure information and its ability to effectively transform information into output features. Based on the concept of MINE, we use discriminators to distinguish between the joint probability distribution and the marginal probability distribution. Specifically, we adopt the Donsker-Varadhan formulation of the KL-divergence to provide a lower-bound of MI:

19 $$ \left( {X,Z} \right)\hat{I}_{{DV}} \left( {X,Z} \right) = E_{{\left( {x,z} \right)\sim p\left( {zx} \right)p\left( x \right)}} \left[ {T_{\omega } \left( {x,z} \right)} \right] - {\text{log}}E_{{\left( {x,z} \right)\sim p\left( z \right)p\left( x \right)}} \left[ {e^{{T_{\omega } \left( {x,z} \right)}} } \right],$$

where ${T}_{\omega }$ is the neural network discriminator parameterized by $\omega$. Then, we refine the encoder $E$ by approximating and maximizing the mutual information. The loss function for global mutual information estimation is:

20 $$Los{s}_{G}=arg\text{max}{\widehat{I}}_{DV}\left(X,Z\right),$$

since our primary objective is to maximize MI rather than obtain specific values, we adopt an alternative estimation approach, such as the Jensen-Shannon (JS) divergence [35], whose advantage over the KL-divergence is the symmetry and the existence of an upper bound, the formulation is:

21 $${\widehat{I}}_{JS}\left(X,Z\right)={E}_{\left(x,z\right)\sim p\left(z\left|x\right.\right)p\left(x\right)}\text{log}\left[{T}_{\omega }\left(x,z\right)\right]+{E}_{\left(x,z\right)\sim p\left(z\right)p\left(x\right)}\text{log}\left[1-{T}_{\omega }\left(x,z\right)\right].$$

For local mutual information estimation, we focus on the local substructures of the input and their matching local output features. First, we encode the input as a feature mapping: ${C}_{\psi }\left(x\right)={\left\{{C}_{\psi }^{\left(i\right)}\right\}}_{i=1}^{M\times M}$, which denotes the local structure of the input, $M\times M$ denote the number of local regions. The ultimate goal is to enhance the average mutual information shared by these local structures and their respective local features. There are average local MI estimation and loss function:

$${\widehat{I}}_{L}\left({X}^{\left(i\right)},{Z}^{\left(i\right)}\right)={E}_{\left({x}_{i},{z}_{i}\right)\sim p\left({z}_{i}\left|{x}_{i}\right.\right)p\left({x}_{i}\right)}\text{log}\left[{T}_{\omega }^{L}\left({C}_{\psi }^{\left(i\right)}\left(x\right),{z}_{i}\right)\right]+{E}_{\left({x}_{i},{z}_{i}\right)\sim p\left({z}_{i}\right)p\left({x}_{i}\right)}\text{log}\left[1-{T}_{\omega }^{L}\left({C}_{\psi }^{\left(i\right)}\left(x\right),{z}_{i}\right)\right],$$

22 $$Los{s}_{L}=\underset{\omega ,\psi }{arg\text{max}}\frac{1}{{M}^{2}}\sum_{i=1}^{M}{\widehat{I}}_{L}\left({X}^{\left(i\right)},{Z}^{\left(i\right)}\right).$$

A prior mutual information estimation is employed to assess the relationship between the output feature distribution and the prior distribution. For desirable latent representations, it is essential to not only retain the maximum amount of original information but also to ensure that the representation distribution closely approximates a specific prior distribution. In our study, we specify the latent distribution to follow Gaussian distribution. First, under the assumption that $q\left(z\right)$ adheres to Gaussian distribution, we estimate the divergence $D\left(q\left(z\right)\Vert p\left(z\left|x\right.\right)\right)$ by training a discriminator ${D}_{\phi }$, then train the encoder to make $p\left(z\left|x\right.\right)$ approximate $q\left(z\right)$ so as to minimize this estimate. The loss function for prior mutual information estimation is as follows:

$$Los{s}_{P}=\underset{\psi }{arg\text{min}}\underset{\phi }{arg\text{max}{\widehat{D}}_{\phi }}\left(q\left(z\right)\Vert p\left(z\left|x\right.\right)\right)$$

23 $$={E}_{z\sim q\left(z\right)}\text{log}\left[{D}_{\phi }\left(z\right)\right]+{E}_{x\sim p\left(x\right)}\text{log}\left[1-{D}_{\phi }\left({E}_{\psi }\left(x\right)\right)\right].$$

The above three loss functions of mutual information estimation impose constraints on the encoder from global, local and prior perspectives, respectively. By integrating these functions, we obtain the final loss function for mutual information estimation as follows:

$$Los{s}_{MI}=\underset{{\omega }_{1},\psi }{arg\text{max}}\alpha {\widehat{I}}_{JS}\left(\widetilde{A},Z\right)$$

$$+\underset{{\omega }_{2},\psi }{arg\text{max}}\frac{\beta }{{M}^{2}}\sum_{i=1}^{M}{\widehat{I}}_{L}\left({\widetilde{A}}^{\left(i\right)},{Z}^{\left(i\right)}\right)$$

24 $$+\underset{\psi }{arg\text{min}}\underset{\phi }{arg\text{max}\gamma {\widehat{D}}_{\phi }}\left(q\left(z\right)\Vert p\left(z\left|a\right.\right)\right).$$

where the input is flattened and denoted as $\widetilde{A}$, the output feature is represented in a flattened form $Z$, and $\alpha$, $\beta$, $\gamma$ are hyperparameters to balance the loss.

### Overall loss function

The overall loss function comprises the reconstitution loss, spatial topological consistency loss function and mutual information estimation loss function, as follows:

25 $$Los{s}_{total}=Los{s}_{reconstitution}+Los{s}_{STC}+Los{s}_{MI}.$$

Generally, a lower value of the loss function indicates a better-performing model.

## Experiments

### Datasets

We adopt the two heterogeneous biological datasets by Luo et al. [36] and Li et al. [20] in this study. These datasets comprise four node categories and six association types, statistical details are provided in Table 1. In addition, the drug chemical similarity derived from drug chemical structure is quantified with the Tanimoto coefficient, the target sequence similarity is constructed via the Smith-Waterman algorithm.

**Table 1:** Statistics information of Luo’s dataset and Li’s dataset

Type	Items	Luo’s dataset		Li’s dataset	
		Numbers	Resources	Numbers	Resources
Node	Drug (D)	708	DrugBank (3.0) [37]	2214	DrugBank (5.1.8) \* MERGEFORMAT [41]
	Target (T)	1512	HPRD (Release 9) [38]	1968	UniProtKB (2021) \* MERGEFORMAT [42]
	Disease (I)	5603	CTD (2013) [39]	7205	CTD (2021) \* MERGEFORMAT [43]
	Side effect (S)	4192	SIDER (Version 2) [40]	3935	SIDER (Version 4) \* MERGEFORMAT [44]
Edge	Drug-Target	1923	DrugBank (3.0) [37]	8750	DrugBank (5.1.8) \* MERGEFORMAT [41]
	Drug-Drug	10,036	DrugBank (3.0) [37]	1,091,870	DrugBank (5.1.8) \* MERGEFORMAT [41]
	Drug-Disease	199,214	CTD (2013) [39]	542,970	CTD (2021) \* MERGEFORMAT [43]
	Drug-Side effect	80,164	SIDER (Version 2) [40]	104,629	SIDER (Version 4) \* MERGEFORMAT [41]
	Target-Target	7363	HPRD (Release 9) [38]	456,592	STRING (11.0) [45]
	Target-Disease	1,596,745	CTD (2013) [39]	2,922,064	CTD (2021) [43]

### Evaluation metrics

The five-fold cross-validation is adopted to appraise the efficacy of GCNMM. The fundamental principle is to partition the dataset into disjoint subsets, which are alternately utilized as training and test sets. Specifically, all known DTIs are designated as positive samples and split into five equal disjoint subsets through a randomization process. Meanwhile, unknown DTIs are considered as negative samples and undergo the same operation. In each fold, each subset is rotated as the test set while the remaining four subsets are utilized for training.

The assessment metrics include the area under the receiver operating characteristic curve (AUC), the area under the precision-recall curve (AUPR), Recall, F1-score, and Accuracy (ACC).

### Parameter settings

**Fig. 4:**
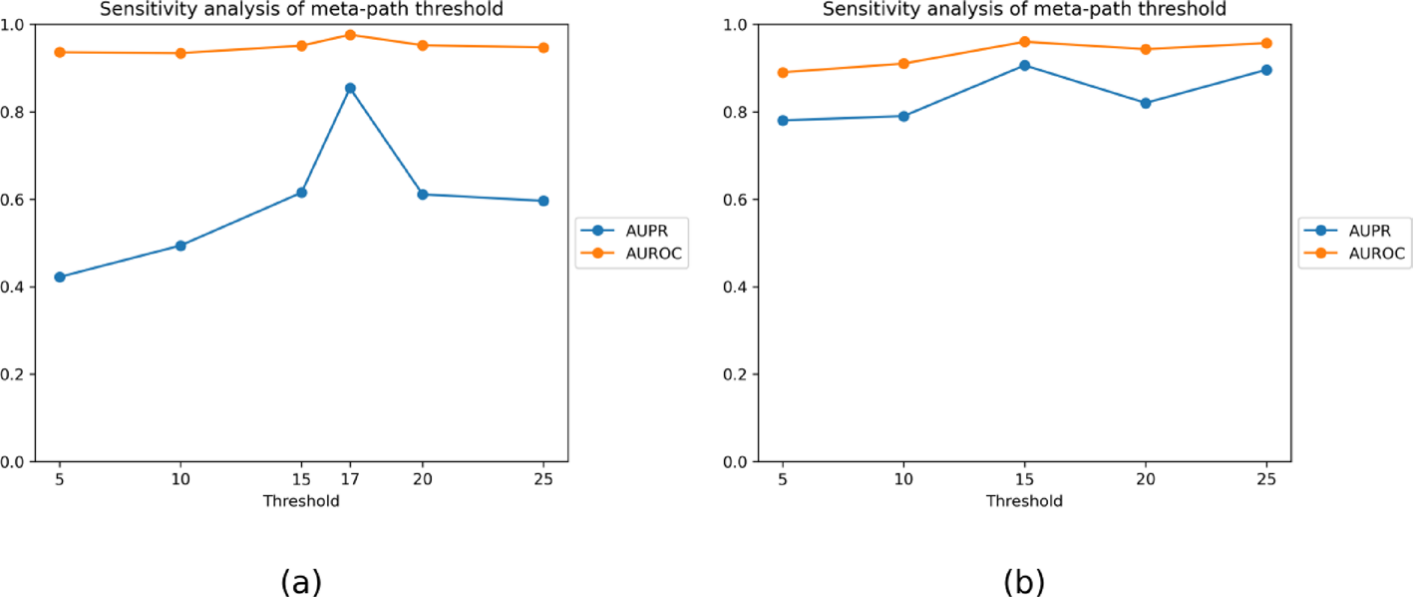
(**a**) The evaluation of different thresholds of the ${A}_{pat{h}_{-}fusion}$ on Luo’s dataset. (**b**) The evaluation of different thresholds of the ${A}_{pat{h}_{-}fusion}$ on Li’s dataset

The GCNMM model was developed with Pytorch 2.1.2 and trained on a Tesla P40 24 GB*4 GPU. The hyperparameters configuration is as follows:

In the fused meta-path network block, the threshold $\tau$ impacts the model performance significantly, as shown in Fig. 4, Luo’s dataset obtains optimal performance at threshold $\tau =17$, while Li’s dataset performs best at $\tau =15$ after tuning the hyperparameters to optimize performance.

**Fig. 5:**
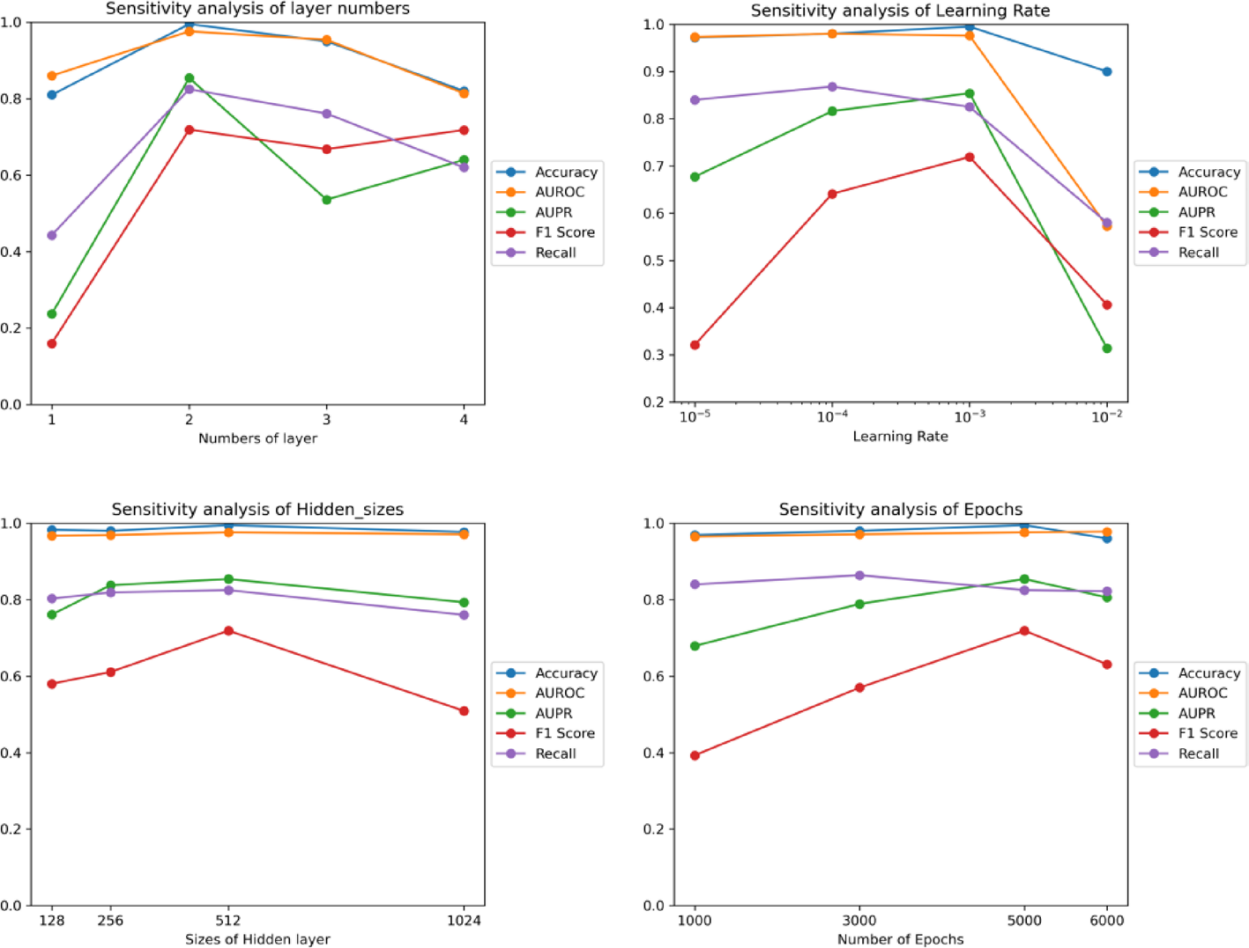
The impact of layer numbers, learning rates, hidden sizes and epochs on Luo’s dataset

**Fig. 6:**
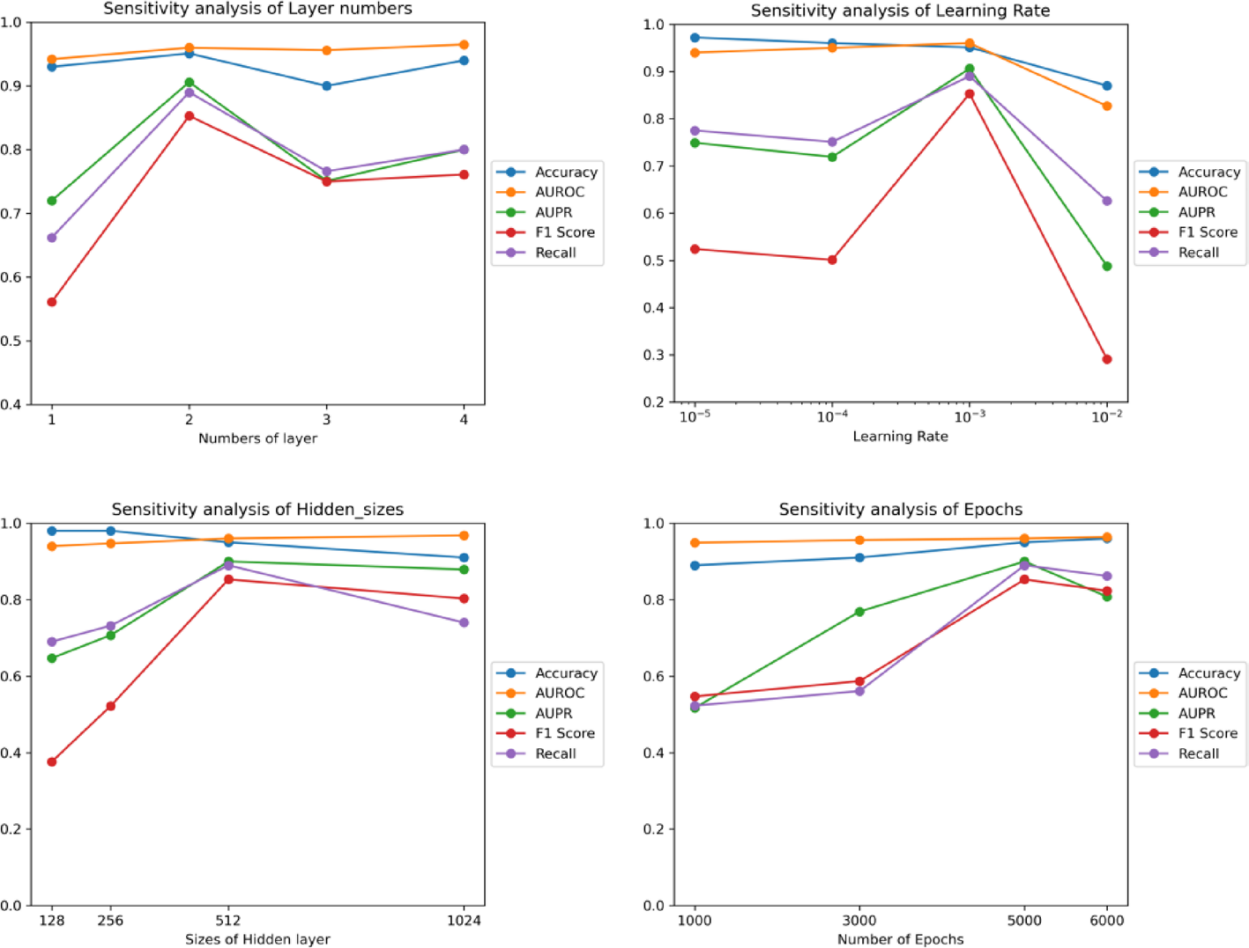
The impact of layer numbers, learning rates, hidden sizes and epochs on Li’s dataset

In the GCN encoder block, a set of parameters is configured to optimize the model performance, as shown in Figs. 5 and 6. After experiments and comparisons of GCN layer $l\in \left\{1,2,3,4\right\}$,$learning rate\in \left\{0.00001,0.0001,0.001,0.01\right\}$, hidden sizes $m\in \left\{128,256,512,1024\right\}$ and $epochs\in \left\{1000,3000,5000,6000\right\}$, ultimately, the learning rate is configured at 0.001, the dimensions of the hidden layer and output are set to 512, 256, respectively. The training batch size is determined to be 64, and the process is conducted over 5000 epochs.

The selection of hyperparameters for the subsequent modules is guided by the ranges suggested in the literature [24, 35]. In the loss function of spatial topological consistency, we set ${\lambda }_{1}$=0.001, ${\lambda }_{2}$=0.001 and ${\lambda }_{3}$=0.01. In the loss function of mutual information estimation, the parameters $\alpha$=0.001, $\beta$=0.001 and $\gamma$=0.0001.

The hyperparameters of classifiers are determined through grid-search, detailed hyperparameters are provided in Supplementary Materials.

### Classifier comparison

The feature vectors are obtained after network representation learning and optimization. Several widely used classification algorithms are adopted to make predictions and evaluate performance, including LightGBM (LGB), Categorical Boosting (CatBoost), Multilayer Perceptron (MLP), Logistic Regression (LR), Adaptive Boosting (AdaBoost), Random Forest (RF), and eXtreme Gradient Boosting (XGBoost).

**Fig. 7:**
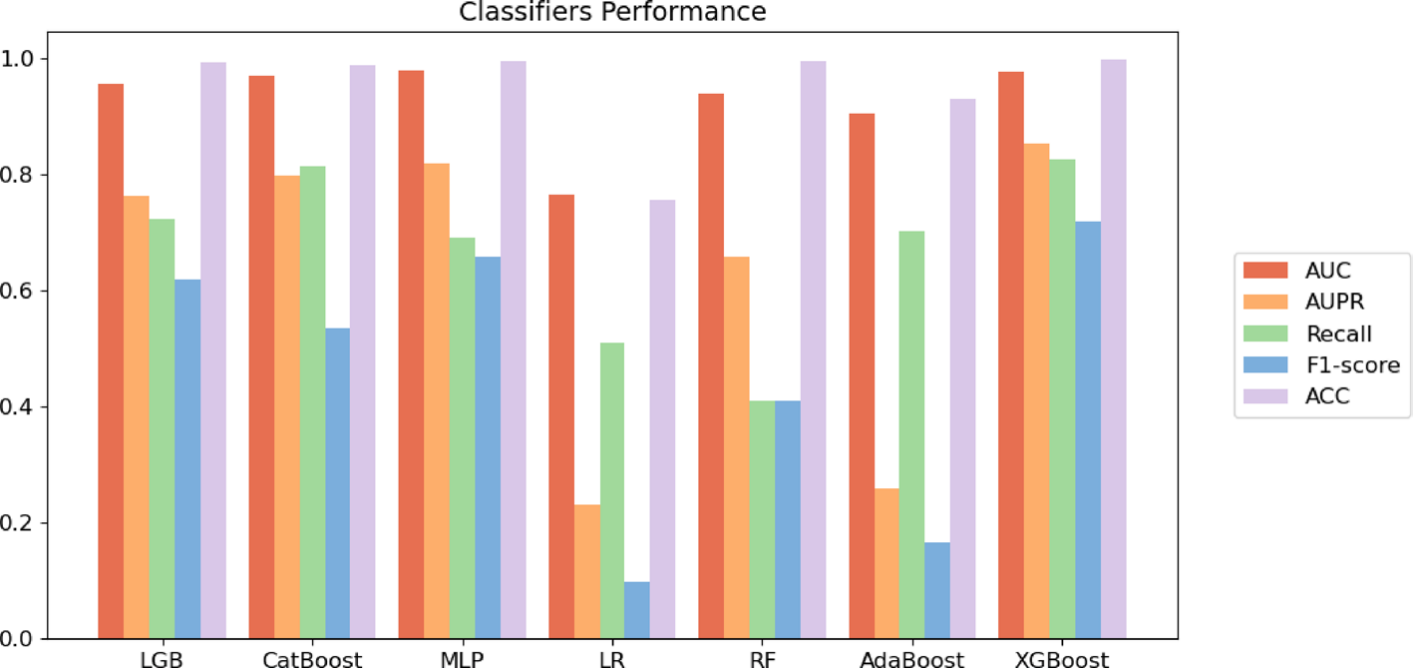
Comparison of different classifiers on Luo’s dataset

**Fig. 8:**
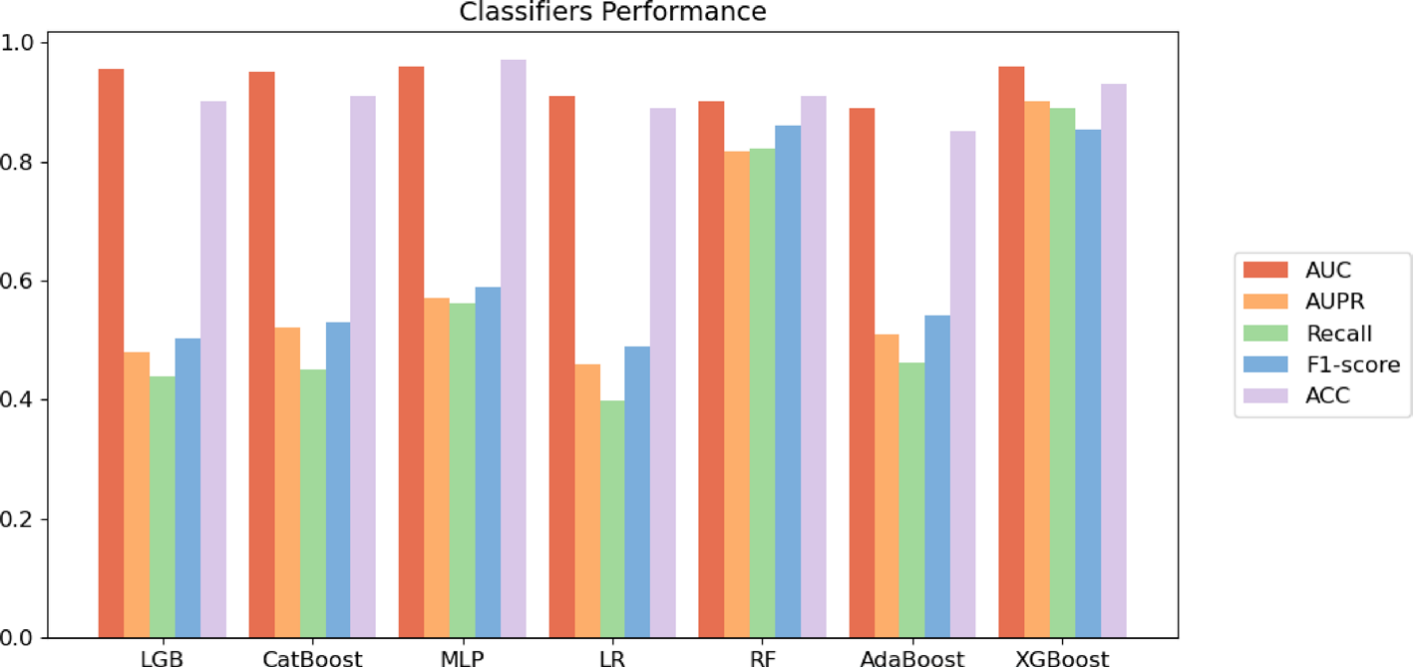
Comparison of different classifiers on Li’s dataset

Each classifier performs five-fold cross-validation. Figures 7–8 display the average metrics of the aforementioned classifiers, which indicate that the XGBoost classifier surpasses the other classifiers. Although the MLP classifier has a marginally higher AUC value than the XGBoost classifier, the XGBoost classifier demonstrates superior overall performance. Consequently, we select the XGBoost classifier for predicting DTIs.

### Baseline models

To highlight the capability of GCNMM, we select various baseline models for comparison, including GADTI [46], VGAEDTI [47], SDGAE [35], SMGCN [48], MFADTI [49] and NEODTI [50].

- **GADTI** is a graph autoencoder approach that combines GCN with random walk to acquire node embedding, DistMult is utilized as a decoder for the DTI prediction.
- **VGAEDTI** is a DTI prediction approach that integrates variational inference and graph auto-encoder to acquire deep features of drugs and targets, then random forest algorithm is to make prediction.
- **SDGAE** is a GCN encoder that trains the model by using spatial consistency constraint and adversarial model, then predicts DTIs based on LightGBM classifier.
- **SMGCN** fuses multi-similarity and employs GCN to derive multi-layer low-dimensional embedded vector, then incorporates multiple kernel learning to integrate embedding features. Afterwards, the Dual Laplace Regularized Least Squares approach is utilized for DTI prediction.
- **MFADTI** applies graph learning from interaction graph, then a chemical structure learning mechanism is utilized to derive structures features. A multi-head self-attention mechanism as fusion module to combine feature vectors for prediction.
- **NEODTI** is a DTI prediction approach based on convolutional neural networks, it aggregates neighborhood information and conducts topological learning for automatic feature extraction pertinent to drugs and targets in heterogeneous networks.

The outcomes of the comparison experiments with these baseline models on two datasets are shown in Figs. S1-S2 and Table 2, with the best values highlighted in bold. Specifically, GCNMM demonstrates overwhelmingly superior performances across all five evaluation metrics compared to all baseline models on Luo’s dataset, with an AUC of 0.976, an AUPR of 0.854, a Recall of 0.825, an F1-score of 0.719 and an ACC of 0.995. On Li’s dataset, GCNMM achieves the best performances in four out of five evaluation metrics over all baseline models, with notable improvements of 3.78%, 18.43%,11.67%, 25.44% over metrics of AUC, AUPR, Recall and F1-score compared to the best-performing baseline model. While the ACC value of GCNMM is higher than the five baseline models, it is slightly lower than one baseline model (VGAEDTI) on Li’s dataset. Evidently, the GCNMM significantly outperforms the other comparison models in DTI prediction.

**Table 2:** The outcomes of the baseline models on two datasets

Dataset	Model	AUC	AUPR	Recall	F1-score	ACC
Luo’s dataset	VGAEDTI	0.925	0.683	0.688	0.060	0.961
	SMGCN	0.798	0.469	0.600	0.640	0.957
	MFADTI	0.769	0.842	0.729	0.664	0.497
	SDGAE	0.950	0.610	0.679	0.641	0.995
	GADTI	0.881	0.519	0.785	0.182	0.195
	NEODTI	0.917	0.744	0.500	0.597	0.972
	GCNMM	**0.976**	**0.854**	**0.825**	**0.719**	**0.995**
Li’s dataset	VGAEDTI	0.916	0.663	0.669	0.051	**0.956**
	SMGCN	0.782	0.472	0.589	0.588	0.947
	MFADTI	0.679	0.765	0.690	0.665	0.499
	SDGAE	0.925	0.608	0.689	0.376	0.951
	GADTI	0.841	0.354	0.797	0.349	0.221
	NEODTI	0.758	0.359	0.426	0.680	0.840
	GCNMM	**0.960**	**0.906**	**0.890**	**0.853**	0.951

We attribute the outstanding performance of GCNMM to these several modules. First, we introduce meta-path-based networks, which help alleviate the sparsity of the original DTI network. Second, spatial topological consistency ensures that nodes remain close to their neighbors in the embedding space. Third, mutual information estimation strengthens the correlation between the input network and the learned feature representations. Overall, the integration of these modules leads to improved model performance on both datasets.

### Ablation analysis

To demonstrate the rationale of the model components, a series of ablation studies on GCNMM were performed with the following settings:

1. GCNMM: the complete proposed model.
2. GCNMM w./o. Meta: the model without meta-path fusion.
3. GCNMM w./o. STC: the model without spatial topological consistency.
4. GCNMM w./o. MI: the model without mutual information estimation (DIM).
5. GCNMM w./o. Meta STC: the model without meta-path fusion and spatial topological consistency.
6. GCNMM w./o. Meta MI: the model without meta-path fusion and mutual information estimation (DIM).
7. GCNMM w./o. STC MI: the model without spatial topological consistency and mutual information estimation (DIM).
8. GCNMM w./o. Meta STC MI: the model only with GCN module.

**Fig. 9:**
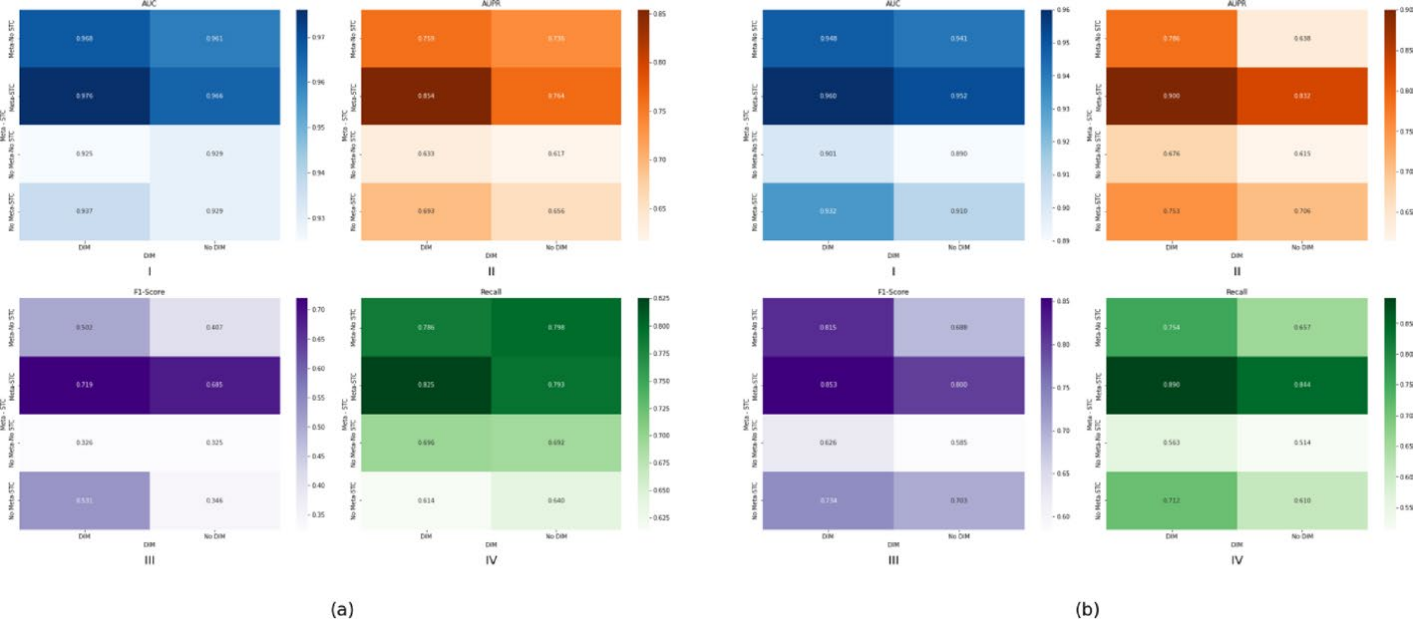
(**a**) The performance of GCNMM and its variants on Luo’s dataset. (**b**) The performance of GCNMM and its variants on Li’s dataset

The model performance metrics (AUC, AUPR, F1-score, Recall) on two datasets are visually summarized through a heatmap representation, where color intensity corresponds to metric superiority, as shown in Fig. 9, the detailed experimental results are shown in Table S1. Specifically, on Luo’s dataset, the AUC, AUPR, Recall and F1-score of the GCNMM w./o. Meta show obvious reductions of 3.99%, 18.74%, 25.58% and 26.15% compared with the original model GCNMM, while on Li’s dataset, the reductions are 2.92%, 16.89%, 20% and 13.95%, indicating that meta-path fusion is crucial in GCNMM. Similarly, spatial topological consistency is essential for model performance. Compared to GCNMM, the AUC, AUPR, Recall and F1-score of the GCNMM w./o. STC on Luo’s dataset show decreases of 0.82%, 13.8%, 4.73% and 30.2%. While on the Li’s, the decreases are 1.25%, 13.25%, 15.28% and 4.45%. Additionally, compared to GCNMM, the results of the GCNMM w./o. MI on Luo’s dataset exhibits reductions of 1.02%, 10.54%, 8.7% and 10.43% in AUC, AUPR, Recall and F1-score, respectively. On Li’s dataset, the reductions are 0.83%, 8.16%, 10.11% and 1.06%, respectively. The ablation analysis results demonstrate that each component contributes significantly to the model GCNMM, underscoring the synergistic efficacy of meta-path fusion, spatial topological consistency, and mutual information estimation.

### Case studies

To additionally assess and verify the predictive capability of GCNMM, three cases are implemented, including two drugs and one target. The two drugs are Methysergide (DrugBank ID: DB00247) and Epinephrine (DrugBank ID: DB00668), and the target is SLC6A4 (UniProt ID: P31645). Methysergide is a drug used for migraine headaches by effectively regulating the diastolic function of cerebral blood vessels, and also for the treatment of carcinoid syndrome. Epinephrine is an endogenous catecholamine used for cardiac arrest, anaphylactic shock, acute bronchial asthma, etc. SLC6A4 is a solute carrier gene mainly involved in the transport regulation of the neurotransmitter serotonin and plays a key role in psychiatric and neurological conditions such as depression, anxiety, and gastrointestinal diseases.

We estimate the likelihood of binding interactions for each selected drug and target across all entries in the dataset and rank them according to their prediction scores. Tables S2–S3 show the top 15 predicted items. Fourteen out of fifteen predicted targets of Methysergide are confirmed, where five predicted targets are the true targets of Methysergide by Luo’s dataset, and the other nine predicted targets are bona fide targets of the drug, as demonstrated in recent studies and public databases. Twelve out of fifteen predicted targets of Epinephrine are confirmed, where seven predicted targets are the true targets of Epinephrine by Luo’s dataset, and the other five predicted targets are known targets of the drug, as demonstrated in recent studies and public databases.

Similarly, the top 40 predicted drugs of SLC6A4 are presented in Table S4. Thirty-seven out of forty predicted drugs are confirmed, where thirty predicted drugs are known drugs of SLC6A4 by Luo’s dataset, and the other seven predicted drugs are bona fide drugs of the target, as demonstrated in recent studies and public databases.

Coach-D server is a molecular docking approach that reflects the binding ability of targets and ligands [51]. Pose^u^ Docking energy and C-Score by Coach-D server are applied to quantify the binding affinity of drugs for targets. Lower Pose^u^ Docking energy values correlate with a more potent binding capacity, reflecting a more stable and favorable interaction. The C-score is used to assess the quality and dependability of molecular docking outcomes, with higher values signifying greater confidence in the ligand’s binding conformation.

**Fig. 10:**
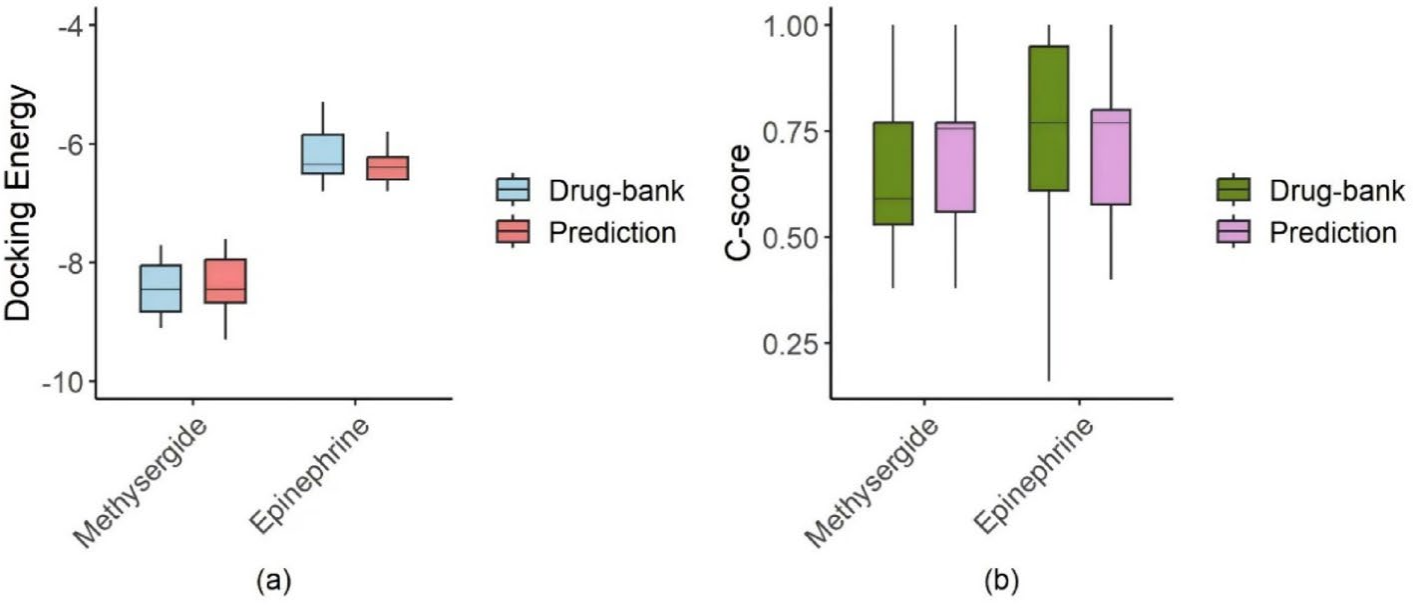
The box plots of molecular docking’s results of selected drugs

Docking energy and C-score values for the selected drugs are obtained from the Coach-D server, and their boxplots are shown in Fig. 10. The median docking energy and C-score values between Methysergide and the top 15 predicted targets are -8.45 and 0.755, respectively. The corresponding median values between Methysergide and known targets in DrugBank are -8.5 and 0.59, respectively. The highest docking energy between Methysergide and known targets is -7.7, indicating that Methysergide can bind to nearly all of the top 15 predicted targets. Similarly, Epinephrine exhibits analogous binding characteristics. The median docking energy and C-score values between Epinephrine and the top 15 predicted targets are -6.4 and 0.77, respectively. The corresponding values between Epinephrine and known targets in DrugBank are -6.3 and 0.77, respectively. The highest docking energy between Epinephrine and known targets is -3.9, indicating that Epinephrine can also bind to nearly all of the top 15 predicted targets.

## Discussion

The exploration of DTIs plays a crucial role in advancing drug discovery. In this study, we propose a new framework called GCNMM. Meta-path networks are fused by using GAT to effectively address the issue of matrix sparsity. The incorporation of spatial topological consistency and mutual information estimation into the GCN encoding process ensures the preservation of topological integrity while strengthening the correlation between input and output features. Comparative evaluations show that GCNMM achieves superior performance over baseline models on two datasets. Ablation studies further confirm the importance of each component—meta-path fusion, spatial topological consistency, and mutual information estimation. Moreover, case studies validate the practical utility of GCNMM in DTI prediction.

Despite these promising results, GCNMM has certain limitations. It relies only on lower-dimensional structural information to obtain direct similarity between items. Additionally, the inclusion of mutual information estimation introduces matrix expansion operations during training, which not only prolongs training time but also increases computational resource demands. Future studies could incorporate three-dimensional structural characteristics of drugs and targets to further enhance model performance. Our subsequent work would focus on optimizing the model architecture, reducing computational complexity, and minimizing resource consumption.

## Conclusion

In this research, we propose a novel graph convolutional network model, called GCNMM, which integrates meta-paths, similarity networks, and dual optimization based on spatial topological consistency and mutual information, to predict novel DTIs from heterogeneous networks. Two datasets were introduced to evaluate the effectiveness of GCNMM. The results of comparative experiments demonstrate that GCNMM achieves superior performance compared to six baseline models across AUC, AUPR, Recall, F1-score, and ACC metrics. In addition, the ablation analysis confirms the rationality of each module. Furthermore, case studies validate the efficacy of GCNMM for DTI prediction, highlighting its potential for drug discovery development.

## Supplementary Information

Below is the link to the electronic supplementary material.

Supplementary Material 1

## Data Availability

The data used in this study were from Luo et al. (10.1038/s41467-017-00680-8) (https://github.com/luoyunan/DTINet) and Li et al. (10.1093/bib/bbac578) (https://github.com/Zora-LM/MHGNN-DTI). The source code is available at https://github.com/Qzj01/GCNMM.

## Abbreviations

DTI: Drug-target interaction
SVM: Support vector machine
GCN: Graph convolutional network
GAT: Graph attention network
MINE: Mutual information neural estimation
SMILES: Simplified molecular input line entry specification
MSE: Mean squared error
STC: Spatial topological consistency
MI: Mutual information
AUC: The area under the receiver operating characteristic curve,
AUPR: The area under the precision-recall curve
ACC: Accuracy
LGB: Lightgbm
CatBoost: Categorical boosting
MLP: Multilayer perceptron
LR: Logistic regression
RF: Random forest
AdaBoost: Adaptive boosting
XGBoost: EXtreme gradient boosting
