# Supplementary material for: Graph convolution network based on meta-paths and mutual information for drug-target interaction prediction
Source: BMC Bioinformatics. 2025 Nov 7;26:275. doi: 10.1186/s12859-025-06295-x (PMC12595897; doi:10.1186/s12859-025-06295-x)
Supplement: Supplementary file 1 — Supplementary Material 1 [file 12859_2025_6295_MOESM1_ESM.docx]

**Supplementary Materials for “Graph Convolution Network Based on Meta-Paths and Mutual Information for Drug-Target Interaction Prediction”**

## Supplementary Figures


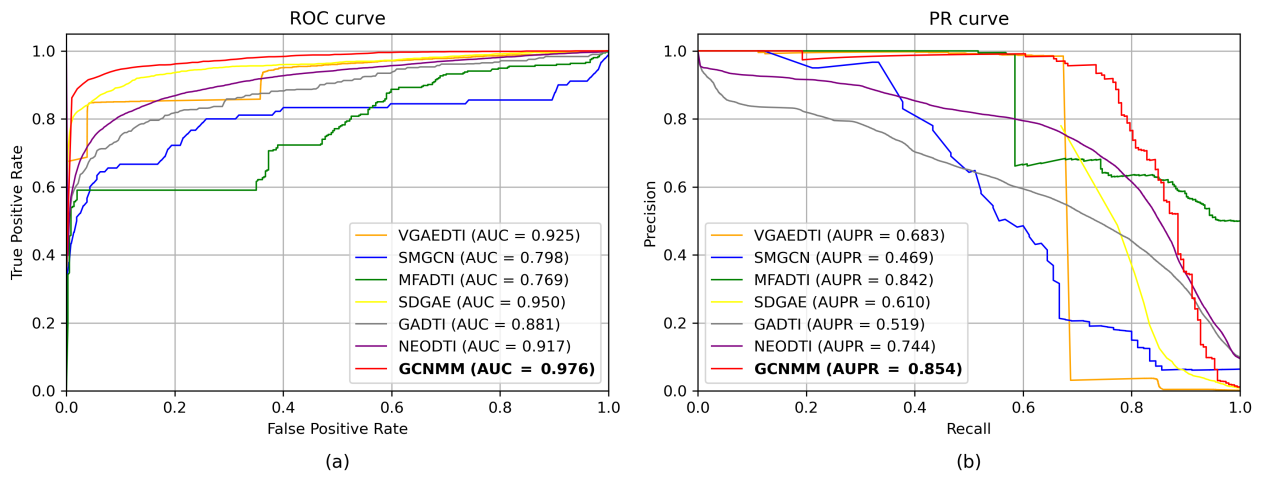


**Fig. S1** The ROC curves and PR curves of baseline models on Luo’s dataset


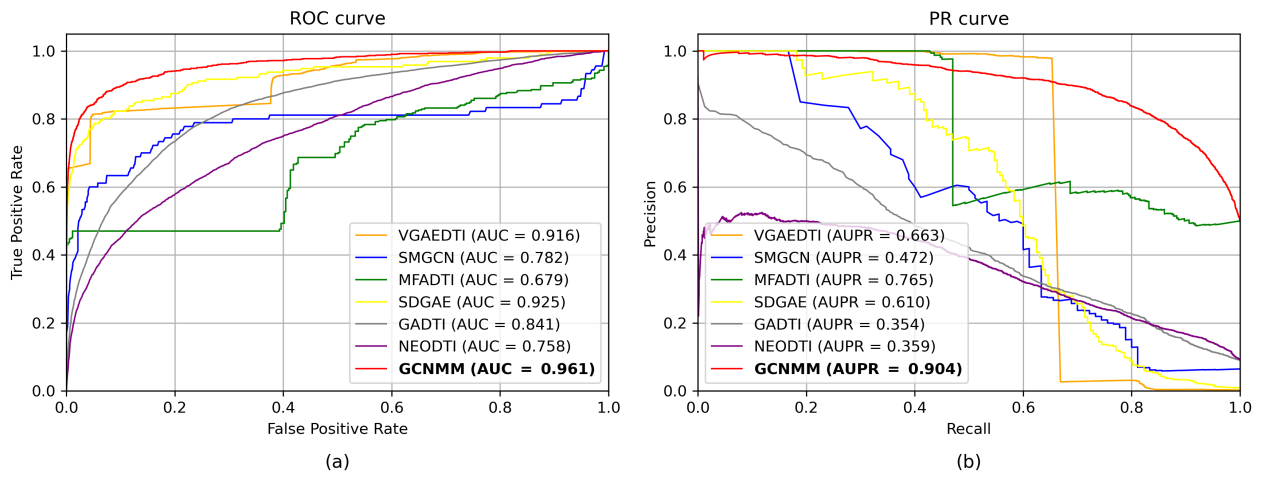


**Fig. S2** The ROC curves and PR curves of baseline models on Li’s dataset

## Supplementary Tables

**Table S1** The ablation results on two datasets

| **Dataset** | **Model** | **AUC** | **AUPR** | **Recall** | **F1-score** |
| --- | --- | --- | --- | --- | --- |
| Luo’s dataset | **w./o. Meta** | 0.937 | 0.693 | 0.614 | 0.531 |
|  | **w./o. STC** | 0.968 | 0.736 | 0.786 | 0.502 |
|  | **w./o. MI** | 0.966 | 0.764 | 0.753 | 0.644 |
|  | **w./o. Meta STC** | 0.925 | 0.633 | 0.696 | 0.326 |
|  | **w./o. Meta MI** | 0.929 | 0.656 | 0.640 | 0.346 |
|  | **w./o. STC MI** | 0.961 | 0.736 | 0.798 | 0.407 |
|  | **w./o. Meta STC MI** | 0.929 | 0.617 | 0.692 | 0.325 |
|  | **GCNMM** | **0.976** | **0.854** | **0.825** | **0.719** |
| Li’s dataset | **w./o. Meta** | 0.932 | 0.753 | 0.712 | 0.734 |
|  | **w./o. STC** | 0.948 | 0.786 | 0.754 | 0.815 |
|  | **w./o. MI** | 0.952 | 0.832 | 0.80 | 0.844 |
|  | **w./o. Meta STC** | 0.901 | 0.676 | 0.563 | 0.626 |
|  | **w./o. Meta MI** | 0.910 | 0.706 | 0.610 | 0.703 |
|  | **w./o. STC MI** | 0.941 | 0.638 | 0.657 | 0.688 |
|  | **w./o. Meta STC MI** | 0.890 | 0.615 | 0.514 | 0.585 |
|  | **GCNMM** | **0.960** | **0.906** | **0.890** | **0.853** |

**Table S2** The top 15 predicted targets of Methysergide

| Rank | Uniprot ID | Target | Result | Evidence |
| --- | --- | --- | --- | --- |
| 1 | P08908 | HTR1A | True | DrugBank |
| **2** | **P28222** | **HTR1B** | **True** | **DrugBank** |
| 3 | P28223 | HTR2A | True | DrugBank |
| **4** | **P08913** | **ADRA2A** | **True** | **Drug Central** |
| **5** | **P14416** | **DRD2** | **True** | **Drug Central** |
| 6 | P41595 | HTR2B | True | DrugBank |
| 7 | P28335 | HTR2C | True | DrugBank |
| **8** | **P21728** | **DRD1** | **True** | **Drug Central** |
| **9** | **P35462** | **DRD3** | **True** | **Drug Central** |
| **10** | P21918 | DRD5 | False | Unconfirmed |
| **11** | **P18089** | **ADRA2B** | **True** | **Drug Central** |
| **12** | **P28221** | **HTR1D** | **True** | **Drug Central** |
| 13 | P34969 | HTR7 | True | DrugBank |
| **14** | **P18825** | **ADRA2C** | **True** | **Drug Central** |
| **15** | **P08684** | **CYP3A4** | **True** | **DrugBank** |

**Table S3** The top 15 predicted targets of Epinephrine

| Rank | Uniprot ID | Target | Result | Evidence |
| --- | --- | --- | --- | --- |
| 1 | P35348 | ADRA1A | True | DrugBank |
| 2 | P35368 | ADRA1B | True | DrugBank |
| 3 | P07550 | ADRB2 | True | DrugBank |
| 4 | P25100 | ADRA1D | True | DrugBank |
| **5** | **P18825** | **ADRA2C** | **True** | **Drug Central** |
| 6 | P08588 | ADRB1 | True | DrugBank |
| 7 | P08913 | ADRA2A | True | DrugBank |
| 8 | Q12809 | KCNH2 | False | Unconfirmed |
| 9 | P18089 | ADRA2B | True | DrugBank |
| **10** | **P14416** | **DRD2** | **True** | **Drug Central** |
| 11 | P28223 | HTR2A | False | Unconfirmed |
| **12** | **P21397** | **MAOA** | **True** | **DrugBank** |
| **13** | **P23975** | **SLC6A2** | **True** | **Drug Central** |
| **14** | Q01959 | SLC6A3 | False | Unconfirmed |
| **15** | **P27338** | **MAOB** | **True** | **DrugBank** |

**Table S4** The top 40 drug candidates which are predicted to interact with SLC6A4

| Rank | DrugBank ID | Drug | Result | Rank | DrugBank ID | Drug | Result |
| --- | --- | --- | --- | --- | --- | --- | --- |
| 1 | DB00476 | Duloxetine | True | 21 | DB00215 | Citalopram | True |
| 2 | DB00285 | Venlafaxine | True | 22 | DB00656 | Trazodone | True |
| 3 | DB00543 | Amoxapine | True | 23 | DB01104 | Sertraline | True |
| **4** | **DB00937** | **Diethylpropion** | **True** | 24 | DB01156 | Bupropion | True |
| 5 | DB01577 | Methamphetamine | True | **25** | **DB00370** | **Mirtazapine** | **True** |
| 6 | DB00193 | Tramadol | True | **26** | **DB00182** | **Amphetamine** | **True** |
| 7 | DB00344 | Protriptyline | True | 27 | DB01114 | Chlorpheniramine | True |
| 8 | DB00458 | Imipramine | True | 28 | DB01364 | Ephedrine | Unconfirmed |
| 9 | DB01105 | Sibutramine | True | 29 | DB00176 | Fluvoxamine | True |
| 10 | DB00422 | Methylphenidate | True | 30 | DB04896 | Milnacipran | True |
| 11 | DB00540 | Nortriptyline | True | 31 | DB00472 | Fluoxetine | True |
| 12 | DB00191 | Phentermine | True | **32** | **DB00924** | **Cyclobenzaprine** | **True** |
| 13 | DB01151 | Desipramine | True | 33 | DB00898 | Ethanol | True |
| 14 | DB00321 | Amitriptyline | True | 34 | DB06204 | Tapentadol | True |
| 15 | DB00715 | Paroxetine | True | **35** | **DB01224** | **Quetiapine** | **True** |
| 16 | DB01242 | Clomipramine | True | 36 | DB00496 | Darifenacin | True |
| 17 | DB01142 | Doxepin | True | **37** | **DB00721** | **Procaine** | **True** |
| 18 | DB01149 | Nefazodone | True | 38 | DB00477 | Chlorpromazine | Unconfirmed |
| 19 | DB00907 | Cocaine | True | 39 | DB00904 | Ondansetron | Unconfirmed |
| 20 | DB00289 | Atomoxetine | True | **40** | **DB00408** | **Loxapine** | **True** |

## Supplementary Notes

## Parameter settings of classifiers

All classifiers are initialized with random seeds fixed at 42 to ensure reproducibility. For the XGBoost classifier, the count of decision trees is set to 1000, the learning rate is initialized to 0.02, the tree depth is 8, and the feature selection ratio is 0.9. For the LGB classifier, the quantity of leaf nodes is established at 80, the learning rate is configured to 0.02, the feature selection ratio is 0.9, the sampling ratio is 1, and the number of iterations is 5. For the AdaBoost classifier, the quantity of weak classifiers is 1000, and the learning rate is fixed at 0.02. For the MLP classifier, the Adam optimizer is configured with a learning rate fixed at 0.001 and weight decay at 0.0001, and the maximum iterations are configured to 1000. For the LR classifier, the optimization algorithm is L-BFGS, the regularization parameter is determined to be 1.0, and the maximum iterations are capped at 1000. For the RF classifier, the quantity of decision trees is established at 1000, the tree depth is set to 8, and the minimum sample size for division and leaf nodes is 2 and 1, respectively. For the CatBoost classifier, the iteration count is 1000, the learning rate is adjusted to 0.02, the tree depth is set to 8, and the weights for positive and negative classes are 10 and 1, respectively.
